# Real-time visuomotor behavior and electrophysiology recording setup for use with humans and monkeys

**DOI:** 10.1152/jn.00262.2017

**Published:** 2018-05-02

**Authors:** Marcel Jan de Haan, Thomas Brochier, Sonja Grün, Alexa Riehle, Frédéric V. Barthélemy

**Affiliations:** ^1^Institut de Neurosciences de la Timone, Centre National de la Recherche Scientifique-Aix-Marseille Université, UMR7289, Marseille, France; ^2^Institute of Neuroscience and Medicine (INM-6) and Institute for Advanced Simulation (IAS-6) and JARA Brain Institute I (INM-10), Forschungszentrum Jülich, Jülich, Germany; ^3^RIKEN Brain Science Institute, Hirosawa, Wako-Shi, Saitama, Japan; ^4^Theoretical Systems Neurobiology, RWTH Aachen University, Aachen, Germany

**Keywords:** arm/hand kinematics, common workspace, eye-hand coordination, eye tracking, perturbation

## Abstract

Large-scale network dynamics in multiple visuomotor areas is of great interest in the study of eye-hand coordination in both human and monkey. To explore this, it is essential to develop a setup that allows for precise tracking of eye and hand movements. It is desirable that it is able to generate mechanical or visual perturbations of hand trajectories so that eye-hand coordination can be studied in a variety of conditions. There are simple solutions that satisfy these requirements for hand movements performed in the horizontal plane while visual stimuli and hand feedback are presented in the vertical plane. However, this spatial dissociation requires cognitive rules for eye-hand coordination different from eye-hand movements performed in the same space, as is the case in most natural conditions. Here we present an innovative solution for the precise tracking of eye and hand movements in a single reference frame. Importantly, our solution allows behavioral explorations under normal and perturbed conditions in both humans and monkeys. It is based on the integration of two noninvasive commercially available systems to achieve online control and synchronous recording of eye (EyeLink) and hand (KINARM) positions during interactive visuomotor tasks. We also present an eye calibration method compatible with different eye trackers that compensates for nonlinearities caused by the system's geometry. Our setup monitors the two effectors in real time with high spatial and temporal resolution and simultaneously outputs behavioral and neuronal data to an external data acquisition system using a common data format.

**NEW & NOTEWORTHY** We developed a new setup for studying eye-hand coordination in humans and monkeys that monitors the two effectors in real time in a common reference frame. Our eye calibration method allows us to track gaze positions relative to visual stimuli presented in the horizontal workspace of the hand movements. This method compensates for nonlinearities caused by the system’s geometry and transforms kinematics signals from the eye tracker into the same coordinate system as hand and targets.

## INTRODUCTION

In natural behavior, humans and animals perform motor actions in response to environmental stimuli. For example, reaching movements are usually triggered by the appearance of an object in the peripheral visual field. This event provokes head and eye movements that bring the image of this object onto the fovea. The visual information is then used to plan and control the coordinated activation of multiple muscles to perform the action. The entire sequence lasts a fraction of a second but involves a large network of cortical (and subcortical) brain structures. These areas work together to coordinate movements of multiple effectors to complete a single goal-directed behavior. When studying eye-hand coordination during such a behavior, it is essential to track the eye and hand positions continuously and precisely to get an understanding of their coordination (Mooshagian et al. 2014; Vercher et al. 1994). The quality of these behavioral measures determines our ability to understand which brain structures are activated and how they interact to perform such tasks, in particular when trying to disentangle their cumulative and independent influence on visuomotor processes (Boussaoud et al. 1998; Cisek and Kalaska 2005; Yttri et al. 2014). Earlier studies suggested that when hand movements involve direct interaction with the object that is being viewed, visuomotor processes rely on a cognitive map classically referred to as “standard mapping” that integrates eye and hand positions in a common reference frame (Wise et al. 1996; see also Archambault et al. 2009; Battaglia-Mayer et al. 2001; Hawkins et al. 2013; Vercher et al. 1994). However, in some specific conditions, eye and hand movements are dissociated, leading to a “nonstandard mapping” (Wise et al. 1996). Nonstandard mapping conditions can occur when hand movements and their visual feedback are in different planes (Archambault et al. 2009), such as a mouse cursor on a screen moving in a different plane than the hand movements on the mouse pad. They can also be produced by the alteration of standard mapping experimental tasks through the application of visual or mechanical perturbations during movement execution by means of virtual reality and robotic devices, respectively (Torrecillos et al. 2014). In all cases, it has been shown that the same task executed in standard and nonstandard mapping conditions generates different patterns of neuronal activity in several brain regions (Connolly et al. 2000; Gail et al. 2009; Gorbet et al. 2004; Granek et al. 2010; Hawkins et al. 2013; Mascaro et al. 2003; Reina and Schwartz 2003; Sayegh et al. 2013, 2014), suggesting that observations made in one mapping condition cannot be extrapolated to the other one.

Although there are simple solutions to record eye movements in the vertical plane while performing horizontal hand movements, there is currently no commercially available solution allowing us to relate eye and hand movements both performed in the horizontal plane that could be applied to human and monkey experiments. In this context, we developed an experimental setup for the continuous recording of eye and hand movements during visually guided motor behavior performed in standard mapping and nonstandard mapping conditions, including visual and/or motor perturbations. Our setup also allowed us to record neuronal activity simultaneously from multiple brain areas along with the behavioral data, and it was designed to be compatible with various electrophysiological recording techniques. Special care was taken to ensure the precise synchronization of the behavioral and electrophysiological recordings. For the purpose of cross-species comparison, we built identical setups for use in monkey and human experiments. In our monkey setup, the system was connected to a device that simultaneously records the activity of multiple single neurons and local field potentials (LFPs), whereas in our human setup it was connected to an electroencephalography (EEG) device. The setups employ similar hardware architectures to accommodate technical demands and run principally identical software relevant for task development, control of behavior, eye calibration, and online data processing.

Asaad et al. (2013) underlined the need to develop tools that are accessible by a large part of the scientific community and compatible with most of the classically used hardware. Indeed, laboratories may develop local solutions to fulfill the needs of a specific project, but these solutions often rely on internal knowledge or skill (e.g., a specific programming language or custom-made pieces of equipment). As a consequence, it could sometimes be difficult to apply them on other projects despite their descriptions in the methods sections of experimental papers. In this report, we propose a solution that can be adapted to multiple hardware configurations. A comparison with existing solutions is provided in discussion.

For both monkey and human setups we selected the KINARM exoskeleton robot because of its ability to record continuous and precise arm/hand movements in the horizontal plane, with the possibility to perturb the movement and its visual feedback (KINARM Exoskeleton Laboratory, BKIN Technologies). The KINARM Exoskeleton Laboratory offers eye tracking as an option in its human version, but this is not available in its nonhuman primate version. For measuring eye movements, the EyeLink system was selected for its infrared noninvasive eye movement recordings (SR Research; EyeLink 1000 for monkeys, EyeLink II for humans). However, these systems come with their own proprietary software and specific hardware features and were designed as independent platforms. To develop an integrated experimental setup, we had to establish a clear hierarchy between the participating systems. We selected the hand tracking system as the master component of the setup, which controls task behavior and timing, motor perturbations and load changes, effector calibration and feedback, visual stimuli, and output of behavioral data. Moreover, experiments in the KINARM system are programmed in MATLAB language (Simulink environment), which is broadly used by experimentalists. The eye tracking system and the data acquisition (DAQ) system (Cerebus, Blackrock Microsystems for monkeys; Biosemi EEG and ADwin Keithley EMG for humans) were integrated as slave components in the setups, meaning that they can be replaced or upgraded without loss of setup functionality. As a consequence of this architecture using a unique master component, both eye and hand movements can be processed synchronously in real time. Finally, we developed a computationally lightweight eye calibration method for both setups to express the position of the gaze in the same (horizontal) coordinate system as the hand positions.

In this report we present in detail the complete integration of the eye tracking system into the hand tracking system for each setup in parallel. In materials and methods, we propose a common solution for each aspect of integration and point out minor setup-specific adaptations when they were necessary. We then present the results of extensive tests designed to assess the reliability of the eye calibration method. The performances of a human participant in the human setup during our tests and those of a custom servo-controlled artificial camera eye in the monkey setup are presented in parallel in results. Once we validated the methods in both setups, we implemented them in real experimental conditions with human and monkey participants engaged in a sequential reaching task.

## MATERIALS AND METHODS

### 

#### Hand/eye movement control systems.

**Fig. 1.**
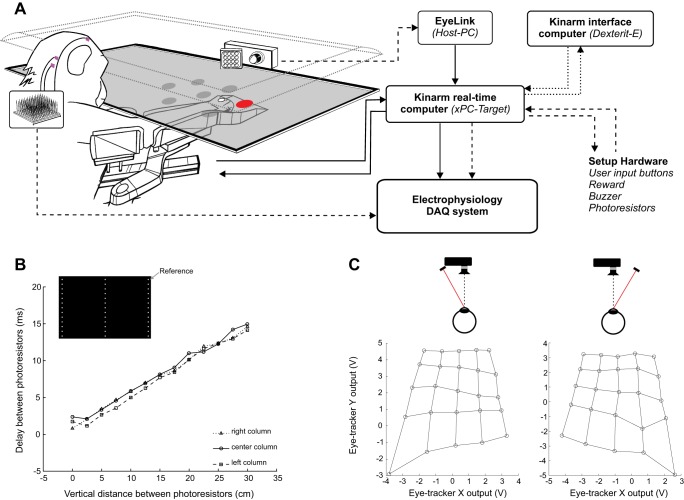
Setup overview and nonlinearities. *A*: organization of the setup with a monkey placed into the exoskeleton, viewing visual stimuli while the camera tracks eye movements. *Right*: analog (solid lines), digital (dashed lines), and Ethernet/UDP (dotted lines) connections between the computers (boxes). *B*: delay between the response peaks of 2 photoresistors to a luminance change on the screen, as a function of the vertical distance between them. *Inset:* possible locations of the probe photoresistor on the screen. Arrow points to the location of the reference photoresistor. *C*: average voltage outputted by the eye tracker on *X* and *Y* channels, when it captures an artificial eye that was aligned to 25 targets located on a 5 × 5 grid, with the infrared light source placed at the right side of the camera (*right*) or at the left side of the camera (*left*).

Both human and monkey setup configurations were built around the KINARM Exoskeleton Laboratory (BKIN Technologies; www.bkintechnologies.com). This recording solution for arm/hand movements in a two-dimensional (2D) horizontal plane was composed of a motorized exoskeleton arm and a virtual reality (VR) display. The system was controlled and operated by two computers (see Fig. 1*A*): the KINARM interface computer (Intel Core i5 760 2.80 GHz, 4 GB RAM), where the operator can program a Simulink model (Simulink Real Time, The MathWorks) and gets control and feedback on the task with the provided software (Dexterit-E, BKIN Technologies), and the KINARM real-time computer (Intel Core 2 duo E8400 3 GHz, 1 GB RAM, DOS-based operating system: xPC-Target, The MathWorks) that runs the compiled Simulink models. The communication between the two computers used a UDP protocol through a direct Ethernet connection.

The motorized KINARM exoskeleton was fixed to a chair, with the upper arm and forearm of the participant placed in arm supports at shoulder height, enabling the arm to move in a horizontal space (Fig. 1*A*). The positions of the joints (shoulder and elbow) were recorded continuously in real time, with two torque motors capable of applying mechanical loads at each joint independently. The hand position was calculated from the joint angles in real time with a trigonometrical reconstruction (KINARM Simulink Library). The chair/exoskeleton module was then fixed to a VR environment, which displayed the visual stimuli and the hand feedback to the participant. The VR display consisted of a horizontal computer screen (Benq VW2420H, resolution 1,920 × 1,080 at 60 Hz) facing downward, with its image reflected on a horizontal semitransparent mirror.

Every image presented on a computer screen is delayed by a latency that is determined by the characteristics of the screen. Before the beginning of the recordings, we therefore evaluated the display latency of our screen, as well as the spatial and temporal properties of its image refresh rate. Two photoresistors were used to record the change in luminance of the screen when it was switched from black to white. One photoresistor was positioned at a constant location (top right corner) of the screen and was used as a reference. The second photoresistor was used as a probe and placed at different locations shown in the screen representation in Fig. 1*B*. The curves presented in Fig. 1*B* show the average latency between the luminance change detection of the two photoresistors as a function of probe location. The delay between the luminance change detection at the reference level and at the probe level increased linearly with the vertical distance following the image refresh direction, from top to bottom of the screen. The temporal dynamic of the vertical drawing of the image was the same for the left side, the center, and the right side of the screen. The observed maximum delay was ~15 ms, which was slightly shorter than predicted by a refresh rate of 60 Hz but coherent if one considers that the photoresistors were not located at the edge of the screen. To control for the precise timing of the visual stimulus (target) presentation during the experiments, target onsets and offsets were accompanied by a change in luminance (e.g., from black to white) of two reference spots on the screen. These two spots (squares, 5 × 5 mm) were located at the top left and bottom right corners of the screen, and their luminances were measured by means of two photoresistors. The signals recorded by these photoresistors were sampled at 1 kHz and stored in the same data file as the behavior. Precise target onset and offset times could therefore be determined off-line according to the space-time function defined above (Fig. 1*B*).

By adjusting the height of the screen and the mirror appropriately, the reflection of the screen images, i.e., the stimuli (targets) and the hand feedback representation, fell into the plane of the hand position (Scott 1999). With internal calibration, the target coordinates and the hand position were expressed in a single reference frame, enabling the setup to react according to the behavior of the participant. The hand underneath the semitransparent mirror could be illuminated, which allowed direct vision of the hand, or obscured and replaced by a projected hand feedback from the task.

For measuring eye movements we employed the EyeLink system (SR Research; https//www.sr-research.com). This noninvasive system uses an infrared (IR) light source directed toward the eye, and the reflection, modified by the eye movement, is captured by an IR camera. To record the participant’s eye movements during a visually guided motor task in either setup, several options were considered to optimize signal quality. In eye tracking configurations requiring high precision, the head of the participant needs to be restrained and the camera should be placed in the center of the visual field. However, the camera would physically occupy the same location as the stimulus. In the monkey setup, using the EyeLink 1000, a classical solution is to place a “hot” mirror (i.e., a mirror that reflects only IR light) in front of the monkey at an angle of 45°. This configuration would allow a camera mounted perpendicular at the side or above the mirror to track the eye, while preserving direct vision of the stimulus (e.g., Reina and Schwartz 2003; EyeLink 1000 Tower/Primate Mount, https://www.sr-research.com/mount_primate.html). However, because of space limitations and the possibility for the monkey to touch the mirror and the camera, use of the standard EyeLink primate mount to set the illuminator position was not possible. We therefore chose to use the direct tracking mode (without a hot mirror) and placed the camera at the back of the VR display (see Fig. 1*A*). In the EyeLink 1000 system, an IR-LED illuminator (light source) flanked the camera and its position relative to the eye determined the location of the corneal reflection used for tracking.

**Fig. 2.**
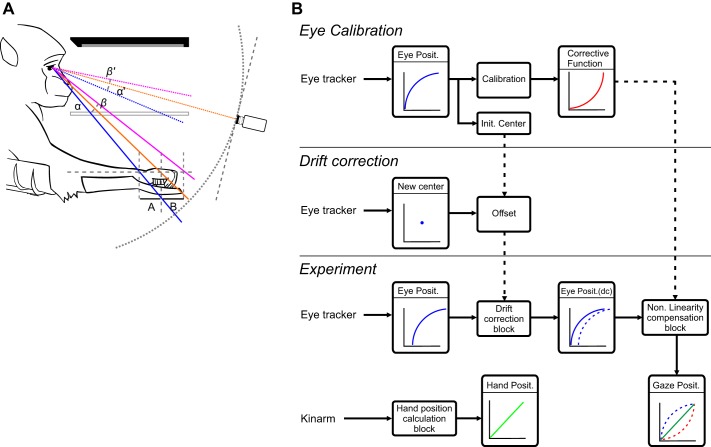
Setup architecture. *A*: nonlinearity introduced by the setup architecture. Three horizontal bars represent from *top* to *bottom* the computer screen (large black bar), the semitransparent mirror (double line), and the display area of the working space (dashed line). *A* and *B* are the distances between the center target and the 2 extreme positions of the targets in depth, having the same length in centimeters. However, visual angles α and β are different, as the angles α′ and β′ seen by the camera, indicated schematically on *right*. *B*: data flow during eye calibration (*top*) and drift correction (*middle*) and during the experiment (*bottom*).

With the camera placed at the back of the VR display, we tested multiple positions of the illuminator and observed the consequences on the eye tracker signal, using an artificial eyeball with a realistic corneal bump and a laser diode to align it to targets. Voltages recorded during target fixation in a grid pattern (18 × 18 cm, aligned with the center of the screen) are shown in Fig. 1*C* for two extreme illuminator positions. The position of the illuminator had a small impact on the signal recorded with the furthermost targets (top lines) but dramatically changed the signal recorded for the closer targets. Because of the viewing perspective with respect to the working space (see Fig. 2*A*), eye movements to capture these targets became larger. In these conditions, the illumination light became tangent to the artificial cornea, and this led to deformations of the corneal reflection spot used by the EyeLink to extract the position of the eye from the camera image. In the most extreme case the spot turned into a line, sometimes with a second reflection point on the sclera leading to a jitter in the eye tracker signal.

In the human setup we used an EyeLink II system. To preserve the possibility of recording EEG in the human setup, access to the head was required; therefore the standard eye tracker helmet that held the two EyeLink II cameras could not be used. Thus the cameras were mounted directly onto the VR display frame with a custom-made forehead and chin support.

The work area of each setup was defined as the overlap of the space the participant could reach in the exoskeleton with the space where the eye tracker was able to record eye movements. The only losses of reaching movements were those very close to the body, where the targets became difficult to view. The work area was 20 × 9 cm in the monkey setup and 28 × 28 cm in the human setup; this difference was mainly due to the longer arm reach of human participants than monkeys. This work area defined the spatial parameters of the task, which was scaled such that all stimuli would fall within it.

#### System integration.

With the prospect of integrating multiple stand-alone systems into a single, precisely synchronized setup, a hierarchy needed to be established. In this context, the KINARM was selected to control the experiment and the eye tracker was used as a sensor to provide the positions and trajectories of the eye. The advantage of such an architecture was that the data flow was centralized in the KINARM real-time computer (xPC Target, The MathWorks; https://www.mathworks.com). An operator controlled the experimental sequence of events and the data collection process in a unique real-time program developed on the KINARM interface computer (Simulink Real-Time, The MathWorks). These programs then ran on the real-time computer, while the operator monitored eye and hand movements and visual stimuli on the screen of the interface computer (Dexterit-E, BKIN Technologies; Fig. 1*A*).

The voltages corresponding to the positions of the eye in the eye tracker image (hereafter referred to as eye position) were collected from two analog outputs of the EyeLink Host PC and sampled in the real-time computer at 1 kHz with an A/D input card (National instruments, PCI-6221). These two analog channels, *X*_v_ and *Y*_v_, expressed the horizontal and vertical deviation of the eye in the eye tracker image, respectively. We chose this option over the possible Ethernet connection between these two computers to avoid interference with the built-in UDP communication between the real-time computer and the interface computer (Fig. 1*A*). Indeed, the UDP protocol does not guarantee communication integrity in this situation. In parallel, the raw exoskeleton motor positions were sampled in the real-time computer at 1 kHz with a motion controller card (Delta Tau Data Systems, PMAC-PCI).

The raw data from the EyeLink Host PC were used to compute the gaze position (i.e., the location on the screen at which the participant is looking) expressed in the same reference frame as the task and the hand position. In this reference frame, the *x*-axis described the position of the gaze (or the target) along the width of the screen. The *y*-axis described the position of the gaze in depth. A single real-time program controlled the interactive components of the task for participants by directly reacting to inputs from the eye tracker and the KINARM exoskeleton. In parallel, the program sent behavioral data continuously to the interface computer for storage in C3D format, together with task parameters. Additionally, the gaze and hand positions were outputted at 1 kHz with a D/A output card (National Instruments, PD2-AO-16x16) to the electrophysiology DAQ system. Although our system was designed with the intention of being compatible with any DAQ system equipped with enough analog and digital channels, here we used DAQ systems that were already in use in our laboratory for monkeys (Cerebus, Blackrock Microsystems; http://blackrockmicro.com; Milekovic et al. 2015; Riehle et al. 2013) and humans (in parallel: ADwin Keithley EMG, https://uk.tek.com and Biosemi EEG, www.biosemi.com; Torrecillos et al. 2014). In both setups, events related to the task sequence (e.g., target onset) and participant behavior (e.g., hand movement onset) were sent as a digital output at 1 kHz by the real-time computer to the setup-specific DAQ systems to ensure the synchronization of the data files and to provide time markers for future analysis. Finally, copies of the raw eye and hand movement data were also sent to the setup-specific DAQ systems.

The integration of these different systems required complex connectivity, and both movement control and DAQ systems required multidirectional connections. Therefore, a custom-made hub was built to regulate communication between these systems, which included multidirectional routed connections and allowed direct operator access to all input/output channels via a front panel with BNC connectors. This hub ensured connections between systems for direct and split signals, with adequate shielding to maintain signal integrity.

#### Eye calibration.

To analyze temporal and spatial features of eye and hand movement behavior in the context of a visually guided arm movement task, all components needed to be expressed in the same reference frame. In the KINARM system, target positions are natively expressed in centimeters in the hand reference frame, which in turn is dependent on the exoskeleton hardware settings. This system offers an integrated VR task environment with high-resolution control of the entire arm; it was therefore deemed simpler to convert the eye tracker signal to fit the hand reference frame than to bring all the KINARM features into the EyeLink system. However, this required a custom eye calibration method that was able to accurately express gaze location in the KINARM task environment and compensate for its nonlinearities introduced by the setup architecture (Fig. 2*A*). The main nonlinearity came from the depth of the work area. Indeed, the use of a horizontal display means that the eye rotation angles (α and β in Fig. 2*A*) were different for two equivalent gaze shifts on the screen (*A* and *B* in Fig. 2*A*) when they were located at different depths. To a lesser extent, this difference also existed between the center and the side of the screen.

As we used the raw signal, no correction was made by the EyeLink software and an unknown supplementary nonlinearity may come from the eye tracker itself (see EyeLink 1000 user manual, section 4.4.2.1). These nonlinearities were highly dependent on the head and eye positions with respect to the screen and the eye tracker camera. This was particularly true in the human setup, where the camera position had to be adapted more often because of the larger group of participants.

To compensate for those nonlinearities that could be subject specific and session specific or that were unknown, we developed an empirical calibration procedure that was self-adapting to the participant’s size and position and to the different setup dimensions (Harezlak et al. 2014; Kasprowski et al. 2014). The goal of the calibration was to establish the transform functions that compensated for spatial distortions by linking the EyeLink eye position signals in volts and the corresponding KINARM-compatible gaze position in centimeters on the screen, on the basis of a target fixation behavior (Fig. 2*B*, *top*).

First, we set the recording range of the eye tracker to fit the size of the work area on the VR display by adjusting the eye tracker gain and offset. In the monkey setup, this was done by directly adjusting the EyeLink 1000 built-in settings. However, in the human setup, we had to build a custom interface to perform these adjustments (offset: ±5 V, gain: ×0 to ×15) on the analog output of the EyeLink II because these settings were not included. This ensured that the eye tracker was able to measure the eye position wherever the participant was looking within the work area, without saturation of the eye tracker output signal and with an optimal use of the signal range. Second, we recorded the *X* and *Y* eye position signals of the EyeLink over 100 ms, while the subject fixated each of the targets (0.2-cm radius) presented in a random sequence on the VR display. We used 25 targets (hereafter referred to as calibration targets) located on a 5 × 5 grid, whose size was adapted to the size of the participants’ work area (see above). These recordings were repeated at least three times to ensure a sufficient amount of data at each target for accurate position estimation.

A graphical user interface (available via https://github.com/INM-6/eye-calibration-GUI) was designed in MATLAB (The MathWorks) to provide a user-friendly interface for calibration of the eye position signal that included trial selection, compensatory model generation, and export of model parameters to a Dexterit-E task file. After trial selection (e.g., after removing blinks and saccades), we computed the average voltages recorded during the different trials for each of the 25 reference points.

**Fig. 3.**
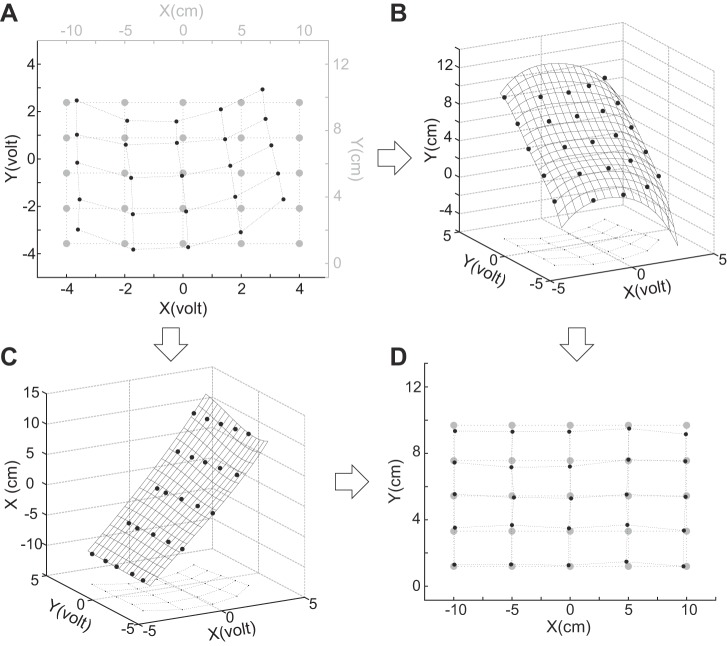
Calibration. *A*: average raw eye position voltages (black dots) at 25 target locations (gray dots). *B*: 3-dimensional (3D) representation of the target position along the *y*-axis of the screen as a function of recorded voltages. The 3D grid is the surface obtained by a biharmonic spline interpolation. *C*: same as *B* for the *x*-axis of the screen. *D*: gaze position on the screen (black dots) reconstructed for fixations on 25 target locations (gray dots).

One typical example of eye position recordings is presented in Fig. 3*A*, scaled to the range of the targets to facilitate the visualization. It clearly shows that the *X*_v_ channel amplitude varies as a function of target position along *X*_cm_ and *Y*_cm_. This dependence on the *X*_cm_ and *Y*_cm_ couple is even more pronounced for the *Y*_v_ amplitude. This result of the setup nonlinearities could be expressed as functions linking each voltage channel with the two screen axes:

$$X_{\text{V}}=f\left(X_{\text{cm}},Y_{\text{cm}}\right)$$

and

$$Y_{\text{V}}=g\left(X_{\text{cm}},Y_{\text{cm}}\right)$$

Consequently, when computing the opposite transformation, we had to consider the gaze position along each screen axis as a function of *X*_v_ and *Y*_v_.

The 25 data points that we recorded during the calibration gave us 25 reference points for the functions *f* and *g*. Because the coordinates of these data points along the *X*_cm_ and *Y*_cm_ axes were the same for the *X*_v_ and *Y*_v_ observed values, we could reverse the relationship and use them as reference points on the functions *f1* and *g1*:

$$X_{\text{cm}}=f1\left(X_{\text{V}},Y_{\text{V}}\right)$$

and

$$Y_{\text{cm}}=g1\left(X_{\text{V}},Y_{\text{V}}\right)$$

The 2D mesh obtained by this axis change is shown in Fig. 3*B* for *X*_cm_ and in Fig. 3*C* for *Y*_cm_. Each grid describes the relationship between the observed values in volts and the theoretical values in centimeters for one of the axes. The consequence of the axis change was to transform regularly distributed data points (in the cm space) into sparsely distributed data points (in the voltage space).

To reconstruct a regularly distributed sample, we adjusted a grid to our data with B-spline interpolation (MATLAB function griddata, using V4 method; Sandwell 1987). The results are shown in Fig. 3, *B* and *C*.

Finally, to generalize the transformation to the whole work area and to make it easily transferable into the Simulink models to be used during experiments, we fit mathematical functions to these grids. We used 2D polynomials because this family of functions can fit to multiple combinations of distortions (see Fig. 1*C*, Fig. 3, Fig. 5, and Fig. 7) and therefore adapt to a large variety of grid shapes. Parameters of the functions are stored in variables in the Simulink task program to adjust its online position conversion module on a daily basis. Figure 3*D* shows the gaze positions reconstructed from the eye positions recorded for the 25 calibration targets.

**Fig. 4.**
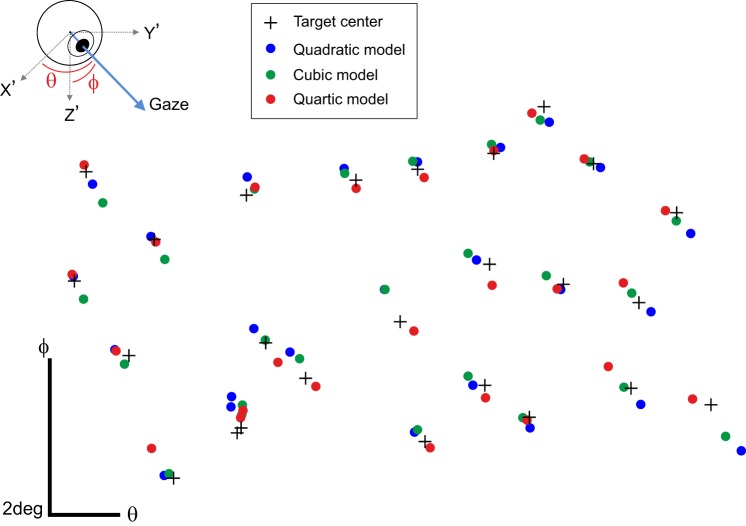
Performance of 3 models to fit multiple combinations of distortions of gaze positions in visual angles for 25 calibration targets presented at random positions: performance obtained with quadratic, cubic, and quartic polynomials. Target and gaze positions are represented in degrees of visual angles θ and φ. *Inset*: cartoon of the gaze position (blue arrow) in a Cartesian 3-dimensional reference frame whose origin was located at the center of the eyeball. *X′*, *Y′*, and *Z′* represent the axes in the eye-based coordinate system.

To define the optimal order for the polynomial function, we made a calibration over the 25-target grid and extracted the parameters of the best-fit quadratic, cubic, and quartic 2D polynomials. The performance of the different models was compared off-line by looking at the gaze positions reconstructed from the same eye positions recorded with 25 circular targets (0.2-cm radius), randomly distributed over the work area. Figure 4 shows the gaze positions in visual angles obtained with the quadratic, cubic, and quartic polynomials, together with the target centers. Average distance to the target was 0.29°, 0.27°, and 0.25° for the quadratic, cubic, and quartic models, respectively. The distances observed with the three models were in the range of the target size. Although the quartic model showed a better accuracy, a paired *t*-test on the measured values returned no significant differences (*P* = 0.46, 0.14, and 0.71 for comparison between quadratic and cubic, quadratic and quartic, and cubic and quartic polynomials, respectively). In the context of this report, we continued with the quartic model.

#### Task participants.

Before applying our eye calibration methods to monkeys and humans, they were first tested and validated with a custom-made servo-controlled artificial eye. This artificial eye was placed at the same location as the monkey’s eye to allow for conservation of hardware settings. It was held by a support that fit in the primate chair and was secured to it to prevent any movement. The artificial eye consisted of a microcamera, which the eye tracker was able to track as a pupil, surrounded by a white plastic disk to mimic a sclera. The camera was mounted on a horizontal axis that could be rotated by a brushless servomotor to move the artificial eye up and down, and the ensemble was mounted on a vertical axis that could be rotated by a second servomotor to produce left-right movements. The two servomotors were controlled by a wireless microcontroller (EZ-B v4 Wi-Fi Robot Controller, EZ-Robot; www.ez-robot.com) which also transmitted a video signal of the eye camera in real time at 20 frames/s in 640 × 480-pixel resolution to a nearby laptop. This provided the operator a visual feedback of the portion of the screen at which the artificial eye was looking. The operator could then make the artificial eye fixate on a target by aligning the cross hairs of the camera with it, using the servomotors, while the eye tracker was outputting the eye position in parallel, as it would have done with a real eye. Since the camera and the plastic disk were not reflective, we could not track a corneal reflection on this artificial eye, and we switched from the EyeLink “pupil + corneal” to “pupil-only” tracking mode, with no negative effects, as the artificial eye was stable enough to not require the corneal reflection measure.

Two human participants (one author, *FB*, and a naive subject) were tested in the human KINARM system with the EyeLink II option (Fig. 3*C*). They were seated with their right arm in the exoskeleton, and their head was stabilized by means of a headrest and a chinrest. All participants were free of known neurological or psychiatric disorders and gave informed consent according to a protocol approved by the Ethics Board of the CNRS (CPP 216-R19).

One female rhesus monkey (*Macaca mulatta*) was used in a sequential pointing task, (*monkey E*; 7 yr, 6.4 kg). All animal procedures were approved by the local ethical committee (C2EA 71, authorization A3/10/12) and conformed to the European and French government regulations. The monkey was kept in a colony of two to four monkeys in a modular housing pen (Allentown, https://www.allentowninc.com), with access to a central play area. It was trained to sit in a modular chair (“Arms Free” monkey chair, BKIN Technologies) by sliding its nylon collar (Primate Products, www.primateproducts.com) into a slightly angled neck plate. The right arm was placed in the exoskeleton, and the left arm was restrained with an L-shaped armlet. To reduce large head movements, a 3D-printed model of the monkey’s head was made from MRI data and used to vacuum form a fitted plastic mask (designed similarly to Slater et al. 2016). This mask was subsequently mounted onto a rigid frame connected to the KINARM chair, and the monkey was trained to position its head into the front half of the mask, while the back half of the mask was attached. Once attached, the entire frame became a rigid structure, with the head fully stabilized.

#### Drift correction.

Because one of the main sources of nonlinearity that we compensated for with our corrective model came from the geometric organization of the setup components (i.e., the eye, the targets, and the camera), a change in head position during task execution would have had a significant impact on the input signal. Indeed, any head movements would lead to a change in the participant’s eye position value in the eye tracker image, causing a global offset in the voltage signal. This offset may cause errors in the reconstruction of the gaze position, through the alteration of the spatial relationship between the signal nonlinearity and the compensatory corrective functions. However, rerunning a complete calibration task because of small unconscious movements of the participant would be time consuming, and detrimental when training a monkey.

A classical solution to this problem is the use of a drift correction, which corrects for the offset of the raw eye tracker voltage by defining and applying a compensatory voltage shift during the experiment. This could be quickly implemented between trials to minimize interruptions of the actual task. The drift correction procedure (Fig. 2*B*, *middle*) consisted of recording the position of the eye when the subject was fixating on a target located at the center position of the grid used during the calibration. The voltages recorded during this fixation were subsequently subtracted from those previously recorded during the calibration. This difference represented the offset introduced by a change in the head position, and this offset was subsequently applied to the raw eye tracker voltage, which brought the signal back into the efficient range of the calibration model.

**Fig. 5.**
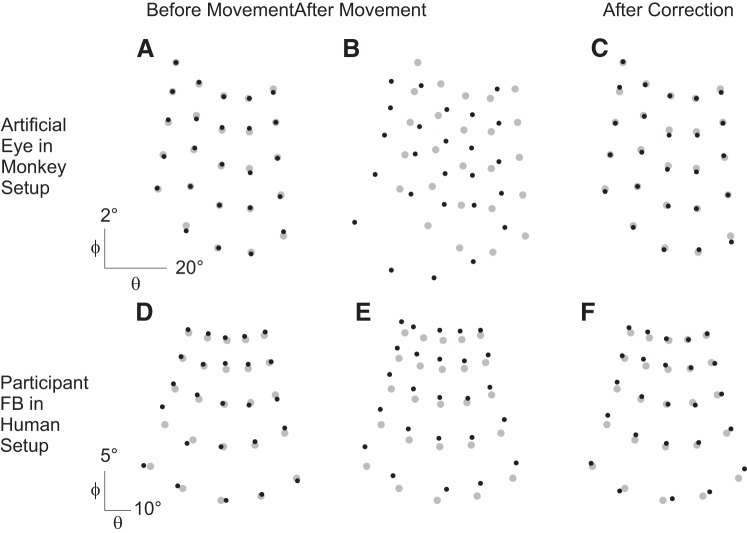
Drift correction. Eye positions (black dots) are shown in visual angles θ and φ (see *inset* in Fig. 4) for 25 targets (gray dots) presented in a 5 × 5 grid arrangement before a head movement occurred (*A* and *D*), after the movement (*B* and *E*), and after drift correction (*C* and *F*) for the artificial eye in the monkey setup (*A–C*) and a human participant (*FB*; *D–F*). This figure shows clearly the efficiency of the drift correction procedure.

**Fig. 6.**
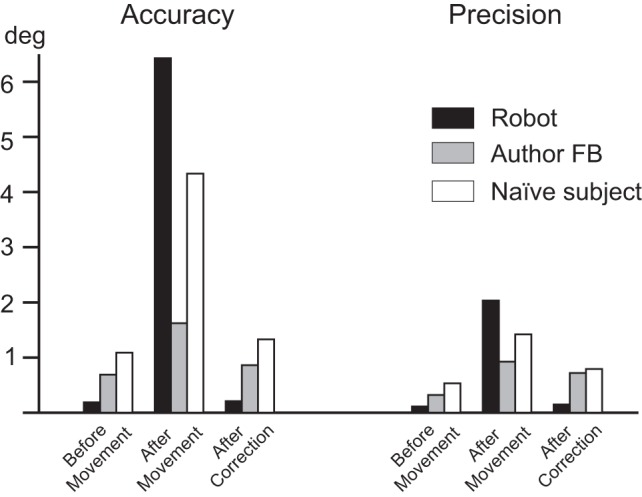
Accuracy and precision before and after drift correction. *Left:* accuracy values (median) obtained during all 3 conditions, i.e., before and after head movement and after drift correction, in degrees for 3 participants, the artificial eye in the monkey setup (Robot) and 2 human participants in the human setup. *Right*: same for precision values (interquartile range).

To test the efficacy of our drift correction, we compared three data sets of reconstructed gaze positions of the artificial eye and human participants during the fixation of 25 validation targets distributed as a 5 × 5-target grid. The first data set was recorded immediately after calibration. This meant that the artificial eye was kept in the same position and that the human participants were instructed to actively maintain the head in a fixed position (before movement: Fig. 5, *A* and *D*, respectively). The second data set was recorded after the position of the artificial eye was moved or the human participant changed his head position (for the artificial eye, its support was moved) (after movement: Fig. 5, *B* and *E*, respectively). Before the third data set was recorded, the artificial eye and human participants had to fixate on a target located at the same coordinates as the center of the calibration grid. The voltage recorded during this fixation was used to calculate the offset required for the drift correction, and this offset was subsequently applied during the recording of the third data set of the artificial eye and human participants (after correction: Fig. 5, *C* and *F*, respectively). Figure 6 summarizes these results by including data of the artificial eye and two human participants. Here we compared the accuracy (median, Fig. 6, *left*) and precision (interquartile range, Fig. 6, *right*) of the distribution of gaze position errors recorded before head movement, after head movement, and after drift correction to establish whether or not the consequence of the artificial eye/head movement visible in the data set recorded after head movement was fully compensated. By using a Wilcoxon rank sum test we confirmed that the data sets recorded before movement and after drift correction had the same distribution (artificial eye: *P* = 0.83; *participant FB*: *P* = 0.43; naive *participant JA*: *P* = 0.38) and were both significantly different from the data set recorded after movement (before vs. after movement: artificial eye/*FB*/*JA*: *P* < 0.01; after movement vs. after drift correction: artificial eye/*FB*/*JA*: *P* < 0.01). Note that we intentionally did not perform these tests with monkeys by introducing voluntarily different head positions, so as not to perturb the training during which a stable head position was required.

#### Gaze position reconstruction.

Figure 2*B*, *bottom*, shows the data flow during the calibration and during the experiment. For calibration, the nonlinearity (represented by a blue curve in the Eye Position box in Fig. 2*B*) of the eye tracker raw signal was used together with the calibration target coordinates to define the parameters of the best corrective function (represented by the red curve in the Corrective Function box in Fig. 2*B*). During the experiment, the first step was to realign the eye tracker raw signal into the range of the recordings made during the calibration by means of the drift correction block. This realigned signal is represented by the shift between the dashed curve and the solid curve in the Eye Position (dc) block in Fig. 2*B*. Once corrected, the signal was converted in a second step by the compensatory function that came from the calibration to obtain the reconstructed gaze position (represented by the green line in the Gaze Position box in Fig. 2*B*). In parallel, the hand position was reconstructed by the built-in KINARM hand position calculation block.

Because the targets and the hand signal are natively expressed in the same reference frame of the screen, the gaze position reconstructed with a set of functions that link the raw eye signal to the target positions also fell into this reference frame, which was outputted in centimeters. This measure can be useful to compare gaze position with hand position. However, it is also important to know the eye position in degrees of visual angle to study eye movements themselves. To retrieve the eye position, we applied a method very similar to that presented in Singh et al. (2016). We first expressed the gaze position in a Cartesian 3D reference frame (hereafter defined as eye reference frame) whose origin is located at the center of the participant’s eyeball (Fig. 4, *inset*). The first dimension (*X′*) varied along a horizontal axis, with positive values on the right side of the eye and negative values on its left side. The second dimension (*Y′*) varied along a second horizontal axis, with positive values ahead from body and negative values for those directed behind the eye. The third dimension (*Z′*) represented height, with positive values above the eyeball and negative values below. In this reference frame, the VR display image was parallel to the *X′Y′* plane and the screen width and screen height were parallel to the *X′* and *Y′* axes, respectively. Knowing the screen location, we could express the gaze position in the eye reference frame by adding the coordinates of the screen reference frame origin expressed in the eye reference frame to the gaze coordinates expressed in the screen reference frame. Because the eyeball was located at the origin of the eye reference frame, these newly obtained gaze coordinates were also the coordinates of the gaze vectors (i.e., the vectors that go from the eye to the gaze position in the eye reference frame). From the vector coordinates, we extract the cosine of the angle by calculating the scalar product of the vectors:

$$\text{cos α}=\frac{\left(\vec{u}\times \vec{v}\right)}{\left(\left\|\vec{u}\right\|\times \left\|\vec{v}\right\|\right)}$$

The angle α expresses the distance in degrees of visual angle between two gaze positions or between one gaze position and a target position. We used this measure to compare, for example, distance distributions. For analysis that required orientation information, the gaze positions in degrees of visual angle were computed by transforming gaze positions from the eye reference frame (*X′*, *Y′*, *Z′*) in centimeters into polar coordinates (ρ, ϕ, θ) in degrees (Fig. 4; Singh et al. 2016).

#### Validation of method.

Each test started with a calibration of the eye position, followed by different validation experiments in which the participants were asked to fixate at individual targets.

To compare the performance of our setup with the industry standard for eye tracking, we used the methods described extensively in Holmqvist et al. (2011). The main measure they proposed is called precision, which expresses the variability of the eye position signal over time. It is defined as the root mean square (RMS) of the angular distances between successive samples recorded during a single fixation trial. This measure is also extensively used by eye tracker companies in their user guides and in the technical description of their systems on their websites (https://www.sr-research.com/EL_1000.html)

By definition, this precision measure is quite insensitive to the low-frequency content in the signal such as that introduced by slow drifts. Moreover, if the recording comes from an artificial eye in a vibration-free environment this measure provides a minimal estimation of the noise level. Although this measure is good for commercial announcements, we estimated that it would not be sufficient to evaluate our system performance in the context of experiments. Indeed, eye movement studies frequently feature average position and/or variability over a large number of experimental trials. However, the precision measured over the average position recorded in successive trials would reflect a variability that could come from the system but could also come from the participant’s behavior or from an interaction between the two. To estimate the performance of our conversion system we compared the distribution of the signal before transformation (input; eye position) with the distribution of the signal after transformation (output; gaze position).

We constructed a normal distribution based on the mean and standard deviation observed in a set of eye positions (in volts) recorded in the monkey for 10 fixations on a single target located at the center of the work area. To observe the impact of the calibration model on the mean and the shape of the distribution we shifted it along the *x*-axis. We calculated both the maximal possible shift of the eye position distribution without becoming significantly different from its original location and the minimal shift needed to become significantly different (Student’s *t*-test; *P* < 0.05 and *P* > 0.05, respectively). The distance between these two means being different or not from the original location was a tenth of the standard deviation of the distribution. These two shifted eye position distributions, and the distribution at the original location, were subsequently put in the gaze reconstruction model, and the gaze position distributions obtained were tested for their mean and shape with a Student’s *t*-test and a Fisher’s *f*-test, respectively. Equivalent analyses were conducted with pairs of distributions shifted along the *y*-axis.

The method for reconstructing the gaze position was subsequently tested over the whole work area with two different target layouts. The first set of 25 targets was located at the same position as the calibration targets, to test the capacity of the model to reconstruct gaze position around calibration reference points. The second set of 25 targets was randomly distributed across the entire calibrated area, to assess the model generalization to the entire work area. For these tests, participants were asked to fixate each target. For each trial, eye position was computed as the average of the samples recorded over 100 ms. To evaluate the quality of the gaze position reconstruction over the work area, we aligned the trials by subtracting the position of the target that was used during the trial from each gaze position. From this position distribution we computed both the precision of the transformation, by calculating the interquartile interval, and the accuracy, by calculating the median of the absolute distance to target across trials.

Finally, to test our calibration model in complete experimental conditions, we recorded the eye and hand positions of a monkey and a human participant performing the same visually guided pointing task toward a sequence of targets. The trial started with the presentation of a circular target (0.2-cm radius) at the center of the work area. The participants were required to reach the target with their hand using the KINARM and to maintain this position for 250 ms to stabilize the hand position at the beginning of the trial. The start target was then turned off, while a second target of the same size and color was presented. This second target was randomly located at one of the vertices of a hexagon centered on the start target coordinates. The participants were then required to reach this new target within 1,000 ms. After staying in the target for 50 ms, the next target appeared at another vertex of the hexagon. Each trial consisted of a sequence of three hand movements, and multiple trajectories were randomized to ensure that no target could be predicted. A trial was considered successful if the participant reached the three targets in the requested delays and with the requested in-target times. It is important to note that eye movements were continuously tracked during the whole sequence but no constraint was applied on eye behavior.

## RESULTS

In the first step of testing our setup we compared its performance with the industry standard for eye tracking and values provided in the literature. In their study, Holmqvist et al. (2011) reported the range for the RMS value, a measure for the variability of the eye position signal over time that could vary with different eye trackers and different types of artificial eye. They found a RMS value of 0.01° for the most precise configurations and up to 1.03° for the poorest. For humans they showed a larger variability of the RMS value due to interindividual differences, but for a given eye tracker they showed that the average of the interindividual RMS distributions corresponded to the value measured with the artificial eye. In our system, the noise was 0.054° with a human participant in the human setup, 0.048° with a monkey, and 0.0054° with an artificial eye in the monkey setup. These values are comparable to the one they reported and also to the one provided by the most commonly used eye trackers (https://www.sr-research.com/EL_1000.html).

As mentioned in materials and methods, we did not consider this test as an adequate indicator of the variability to be expected in our experimental data. Indeed, it is not surprising that our model output does not vary across time because it results from the transformation of a stable input by a continuous mathematical function. For this reason, we tested our gaze reconstruction with a set of inputs that cover a broader range of values that could better reflect what experimental data look like. The goal of these tests was to show that the information present in the eye position was preserved when the gaze position was reconstructed. To test the ability of the system to discriminate gaze positions, we tested the difference between the means of reconstructed gaze position distributions (*n* = 1,000), in case these distributions came from two significantly different eye position distributions or not. Results of the *t*-tests and *f*-tests are shown in Table 1.

**Table 1.** Statistics on generated distributions (P values)

	*t*-Test on θ	*t*-Test on φ	*f*-Test on θ	*f*-Test on φ
Different eye position distributions				
Shift *X*	0.0055	0.4266	0.3786	0.9031
Shift *Y*	0.7214	4.39^−6^	0.9782	0.6168
Same eye position distributions				
Shift *X*	0.1419	0.6869	0.1767	0.815
Shift *Y*	0.908	0.1387	0.977	0.9195

When the compared gaze position distributions came from different eye position distributions (Table 1) a significant difference was shown in the *t*-test on the axis concerned with the shift (i.e., θ for the *x*-axis and ϕ for the *y*-axis). When the compared gaze position distributions came from non-significantly different eye position distributions, the *t*-test showed no significant difference for all shifts. Thus the difference between the means of the gaze position distributions, i.e., after transformation, were only significant for pairs of distributions that came from significantly different eye position distributions, i.e., before transformation. In contrast, two gaze position distributions that resulted from the transformation of non-significantly different eye position distributions showed no significant difference after transformation. Together, these two results clearly show that our gaze position reconstruction preserved the discriminability of eye position samples: two samples that were significantly distinct in the eye position signal remain significantly distinct in the gaze position reconstruction, and two samples that belong to the same distribution in the eye position signal still belong to the same distribution in the gaze position reconstruction.

### 

#### Generalization.

**Fig. 7.**
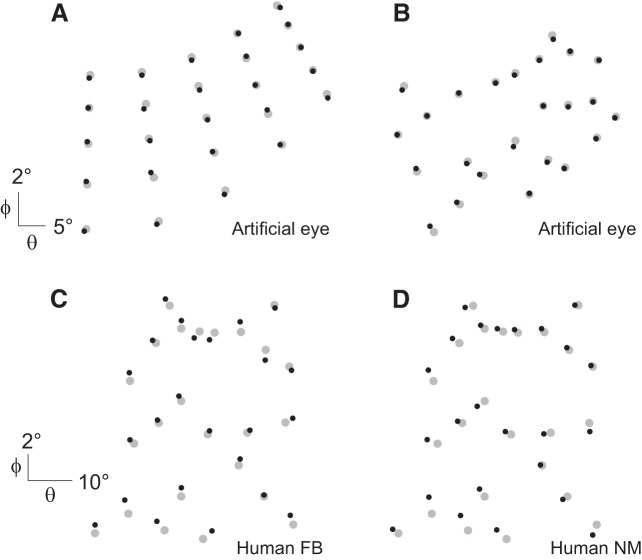
Gaze position reconstructions on multiple targets. *A*: reconstructed gaze positions (black dots) obtained with an artificial eye in the monkey setup for fixations at targets located on a regular grid (gray dots). *B*: same as in *A* but for fixations of targets that were randomly distributed over the work area. *C* and *D*: eye fixations on randomly distributed targets obtained by 2 human participants, *FB* (*C*) and *NM* (*D*).

In the previous tests, we addressed the question of the gaze reconstruction at the level of a single target. By analyzing gaze position reconstructions for fixations of multiple targets scattered over the whole work area, we wanted to address the ability of our system to compensate for nonlinearities in the area covered by the calibration. Figure 7*A* shows the results obtained with an artificial eye in the monkey setup for fixations at targets located on a regular grid. The measured accuracy and precision were 0.26° and 0.17°, respectively. These values are comparable with those shown for drift correction in materials and methods (see Fig. 6, before movement). Because the gaze was reconstructed for fixations at targets located at the same positions as the calibration reference points, it is not surprising to find such small values. Indeed, the artificial eye can almost be considered as an ideal observer, and the differences between the positions recorded during the calibration and those recorded during the tests only came from the steps of the servocontroller. However, these values give a good estimate of the best accuracy and precision one can expect with the artificial eye and provide a baseline to compare them with the measures shown in Fig. 7*B* for fixations of targets randomly distributed over the work area. Accuracy and precision measured on these targets were 0.18° and 0.21°, respectively. We compared these position error distributions with a Wilcoxon rank sum test and found no significant difference between them (*P* = 0.5703). We recorded eye fixations at randomly distributed targets with two human participants [1 author (*FB*) and 1 naive participant (*NM*)] in the human setup (Fig. *7, C* and *D*) and *monkey E* in the monkey setup (Fig. 4, quartic polynomial). Accuracy and precision were respectively 0.70° and 0.60° for *participant FB*, 1.25° and 0.70° for *participant NM*, and 0.23° and 0.2° for *monkey E*. Wilcoxon rank sum tests showed no significant difference when these data were compared to those recorded for the same subject with a regular 5 × 5-target grid (*P* = 0.5823, *P* = 0.9, and *P* = 0.09 for *FB*, *NM*, and *monkey E*, respectively).

**Fig. 8.**
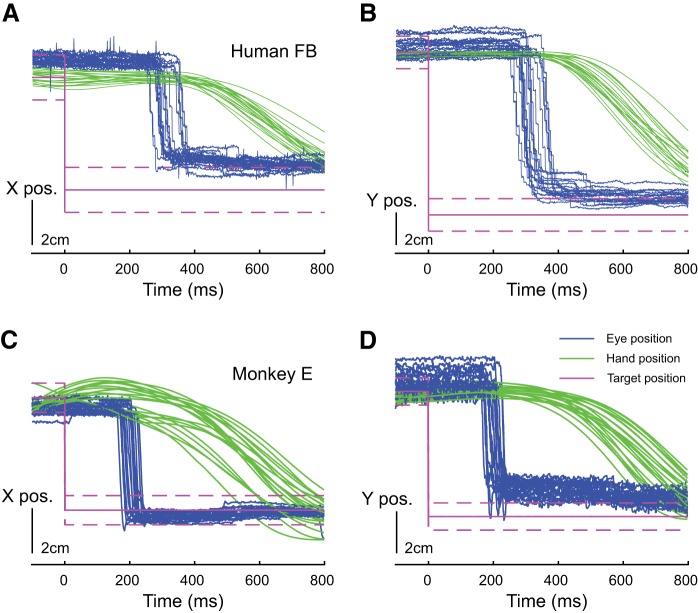
Task-related gaze and hand positions obtained in human *participant FB* (*A* and *B*) and *monkey E* (*C* and *D*), in the *X*-direction (*A* and *C*) and the *Y*-direction (*B* and *D*). Blue traces, eye movements; green traces, hand movements; pink traces, target position; solid line, target center; dashed line, target borders.

Finally, we tested our setup in the context of a hand motor task. Figure 8 shows the gaze position and the hand position along the time axis for 30 recordings during the presentation of the same pair of targets. As the eye behavior was not restrained in this task, some trials had to be removed because they contained saccades outside the work area. However, no filtering or alignment of the traces was performed after data collection. Because multiple trajectories were randomly interleaved during the experiments, the different traces shown in Fig. 8 were separated in time by variable delays from several seconds to several minutes. We can clearly see a consistency of the eye traces across trials, with saccades landing at the edge of the appearing target or showing a small undershoot followed by catch-up saccades that have been extensively described in the literature (Henson 1979; Prablanc et al. 1978). The saccades were initiated after the onset of the targets with latencies of ~200 ms. The hand movement was initiated shortly (~100 ms) after saccade offset. The timing of this behavior is in agreement with the observations of Prablanc et al. (1979) in humans and Rogal et al. (1985) in monkeys. Additionally, the gaze position was sampled 100 ms before the hand entered the target for all trials in order to calculate the overall consistency for human and monkey eye behavior in this particular example task, using the precision measure presented above. This yielded a precision of 0.41° for monkey in Fig. 8*A* and a precision of 0.38° for human in Fig. 8*B*. The gaze position consistency was subsequently calculated for two other targets in repeated trials of the same protocol, yielding a precision of 0.38°/0.60° in *targets 1 and 2* for monkey, respectively, and a precision of 0.19°/0.52° in *targets 1 and 2* for human, respectively.

## DISCUSSION

The primary goal of this work was to develop a system that provides rigorous hand and eye movement control for use in humans and nonhuman primates through noninvasive means, with a high spatial and temporal resolution. To fulfill this goal, we integrated an eye tracking system and a hand tracking system into a unified setup. These setups realize common processing of the data stream from the two systems and ensure the synchronization of the signals. They conserve all features available for hand movement control and make them available to use with the eye position signal. By choosing the KINARM system with the Simulink platform as the core component of our setups, we were also able to implement most of the functionalities required for online analysis of eye movements, such as blink detection and saccade detection based on angular eye position (Singh et al. 2016), and the incorporation of eye behavior-dependent action in the experimental task, such as velocity-based target onset. Moreover, the setups provide an online conversion of the eye positions into the same reference frame as the hand and the task environment. This conversion extends the reactivity of the setup to eye and hand movements, leading to a strong control over eye-hand coordination in the context of the task. For example, in future studies we will be able to observe the impact of perturbations, applied at many levels, from visual stimuli to movement execution, on eye-hand coordination.

### 

#### Hardware integration.

The combination of the different systems into a unified setup required much care in the integration of the different hardware elements. As shown in materials and methods, both camera and light source positions were crucial to ensure a coherent signal wherever the participant looked. In the monkey setup, we chose to install the EyeLink 1000 camera and the illuminator at the back of the VR mirror to keep them out of reach. With this configuration, the corneal reflection was located in the superior half of the eye, near the center. Because of the horizontal screen, the monkey had to look down to perform its task (see Fig. 2*A*), leading the superior eyelid to partially cover the pupil. When the target was located very close in the peripersonal space, the eye was so low that the area of corneal reflection reached the edge of the eyelid, leading to signal instability or even signal loss. The limit for correctly catching eye signals defined the proximal border of the work area. In monkeys this critical area corresponded to a portion of the screen that the monkey was not able to see because of its muzzle. In humans, with the usage of the same camera configuration, the eyelid covering the corneal reflection spot was a more important limitation. Indeed, the subsequent signal loss occurred at screen locations that the participant was perfectly able to see. Thus the impact on the work area could not be neglected. In their human gaze tracker, BKIN Technologies use the VR display mirror to illuminate the eye from below. We have chosen a different approach by using an EyeLink II eye tracking system with a camera mounted close to the eye that records from below. This system allows the participant to look further down without any loss of the signal, preserving the bottom edge of the work area. The top edge was not affected by the introduction of the eye tracking system into the setup, and the work area was only limited by the reach of the participant.

#### Software environment.

Our system was designed around the KINARM system, integrating the signal of an eye tracker as an input to the Simulink model that was in charge of the real-time control over the experiment. Multiple third-party solutions exist to manage experimental setups, such as PsychToolbox (Brainard 1997), MonkeyLogic (Asaad et al. 2013) based on MATLAB language, or PsychoPy (Peirce 2007) based on Python. They all provide complete toolsets for eye movement collection but no direct integration of the KINARM exoskeleton. The use of one of these solutions would have required an extra computer and would have increased the complexity of the communication. As the KINARM system already provided an open programming environment, we had the possibility to integrate the EyeLink signal directly into it.

In this report, we present the results of multiple tests designed to estimate the quality of our reconstructed eye position signal. The recordings made in an ideal situation with an artificial eye or with a human participant showed that our conversion system is very efficient in terms of accuracy and precision. Indeed, our results are comparable with the native accuracy and precision given for commercially available eye tracking solutions in humans (see specification in www.bkintechnologies.com/bkin-products/kinarm-exoskeleton-lab).

We also showed that our gaze position reconstruction preserved the information contained in the recorded eye position without increasing the noise level of the data (Fig. 6). However, the model did not compensate for the input variability. As a consequence, the quality and the stability of the input signal are essential for the accurate and precise estimation of gaze location.

A predictive model could be more efficient to automatically compensate for changes in the signal but would limit the possibilities for unrestrained eye movements. In the recordings we made during a visually guided target tracking task, we showed that arm movements did not perturb the signal stability within a trial (see Fig. 8). Consequently, the periodic drift assessment and correction should be efficient enough to compensate for head movements in normal recording conditions in humans. This drift correction (see Figs. 5 and 6), together with the flexibility of the nonlinearity compensation model (see Fig. 4), allows for the use of a mask rather than a head post in the monkey experiments. Here the use of a mask as a noninvasive solution to stabilize the head was already reported (Fairhall et al. 2006; Slater et al. 2016). These solutions present many advantages over the classical implantation of a head post, the first being the reduction of surgical risks. In the context of multielectrode array (MEA) implantation, the absence of a head post saves space on the animal’s skull for connectors. If electrophysiological data are not required and a completely head-free condition is essential, head tracking may offer an alternative to a mask, as it is capable of offering a higher stability than just pupil and corneal reflection tracking. However, these head tracking solutions would require additional steps in calibration that add to the complexity of training a monkey and would have to be adapted from human head tracking solutions. As far as we know, no specialized monkey head tracking system currently exists.

With the advent of chronic high-density MEAs, investigations of the neural mechanisms of parallel processing architectures in the cerebral cortex reached a milestone in the 1990s. In particular, a seminal research report outlined the recording capabilities of the 100-microelectrode “Utah” array (Campbell et al. 1991; Nordhausen et al. 1996). Since then, extracellular recording technologies have continued to expand, covering larger cortical surfaces with greater spatial resolution and sustainable recording quality (Charvet et al. 2010; Chase et al. 2012; Cheung 2007; Crist and Lebedev 2008; Fernández et al. 2014; Nicolelis et al. 1997; Kelly et al. 2007). Multiple MEAs have been implanted in several cortical areas of the monkey (e.g., Michaels et al. 2015; Poort et al. 2012; Takahashi et al. 2015), while the growing research in the field of neuroprosthetics benefited from multiple MEAs implanted in motor cortex of paraplegic human patients (Collinger et al. 2014). With the ability to record eye and hand behavior synchronously with massive parallel electrophysiological data, our setup is an efficient tool to study the neuronal mechanisms underlying eye-hand coordination. Importantly, this configuration allows us to fully control what a subject sees and how a subject moves. For example, the setup is built to have hand and eye feedback in the same coordinate reference frame in order to examine standard mapping; however, the hand feedback can be decoupled from the hand movement by applying a gain or rotation on the feedback, thus creating a nonstandard mapping condition (Battaglia-Mayer et al. 2001; Wise et al. 1996). Alternatively, the visual feedback of the hand remains intact, but the hand movements are affected by predefined or task-related motor perturbations (e.g., viscosity or local force fields; Torrecillos et al. 2014). These attributes can be used in conjunction or separately or entirely neglected depending on the requirements of the task. The efficient online treatment of signals provided by the setup and its flexibility in terms of input/output connectivity makes it a serious candidate for further development to support a brain-machine interface, whereby electrophysiological and behavioral data are not just recorded but have a direct influence on the progression and outcome of a task (e.g., Carmena et al. 2003; Serruya et al. 2002). Furthermore, the development of this setup for both human and monkey opens promising prospects for translational approaches connecting the monkey model and multiple fields of human research. For example, comparing high-level visual behavior between primate species could be critical in determining whether knowledge gained from animal models is translatable to humans (Rajalingham et al. 2015), and through electrophysiological recordings one could determine whether the same or different areas of visuospatial processing are present in human and monkey (Vanduffel et al. 2002). As a final example, we could determine neural representations of movement parameters by recording monkeys in the monkey setup and implement these parameters into a brain-machine interface of the human setup (Ganguly and Carmena 2009), allowing results from one setup to determine the task parameters of the other one, and vice versa.

## GRANTS

Funding was provided by Deutsche Forschungsgemeinschaft Grant DE2175/2-1 Priority Program (SPP 1665), Helmholtz Portfolio “Supercomputing and Modeling for the Human Brain”, European Union’s Horizon 2020 Framework Program for Research and Innovation under Grant Agreement N° 720270 (Human Brain Project SGA1, SGA2), and Associated International Laboratory “Vision-for-Action” between Centre National de la Recherche Scientifique and Forschungszentrum Jülich.

## DISCLOSURES

No conflicts of interest, financial or otherwise, are declared by the authors.

## AUTHOR CONTRIBUTIONS

M.J.d.H., S.G., A.R., and F.V.B. conceived and designed research; M.J.d.H. and F.V.B. performed experiments; M.J.d.H. and F.V.B. analyzed data; M.J.d.H., A.R., and F.V.B. interpreted results of experiments; M.J.d.H. and F.V.B. prepared figures; M.J.d.H. and F.V.B. drafted manuscript; M.J.d.H., T.G.B., S.G., A.R., and F.V.B. edited and revised manuscript; M.J.d.H., T.G.B., S.G., A.R., and F.V.B. approved final version of manuscript.
